# A Comprehensive Analysis of the Efficacy of Resveratrol in Atherosclerotic Cardiovascular Disease, Myocardial Infarction and Heart Failure

**DOI:** 10.3390/molecules26216600

**Published:** 2021-10-31

**Authors:** Pema Raj, Sijo Joseph Thandapilly, Jeffrey Wigle, Shelley Zieroth, Thomas Netticadan

**Affiliations:** 1 Canadian Centre for Agri-Food Research in Health and Medicine, Winnipeg, MB R2H 2A6, Canada; praj@sbrc.ca; 2 Agriculture and Agri-Food Canada, Winnipeg, MB R3C 1B2, Canada; sijo.joseph@agr.gc.ca; 3 Department of Biochemistry and Medical Genetics, University of Manitoba, Winnipeg, MB R3E 0J9, Canada; jwigle@sbrc.ca; 4 Institute of Cardiovascular Sciences, St. Boniface Hospital Albrechtsen Research Centre, Winnipeg, MB R2H 2A6, Canada; 5 Department of Physiology and Pathophysiology, University of Manitoba, Winnipeg, MB R3E 0J9, Canada; szieroth@sbgh.mb.ca; 6 Section of Cardiology, Department of Medicine, University of Manitoba, Winnipeg, MB R3T 2N2, Canada

**Keywords:** resveratrol, atherosclerosis, myocardial infarction, heart failure

## Abstract

Atherosclerosis, myocardial infarction (MI) and heart failure (HF) are the main causes of mortality and morbidity around the globe. New therapies are needed to better manage ischemic heart disease and HF as existing strategies are not curative. Resveratrol is a stilbene polyphenolic compound with favorable biological effects that counter chronic diseases. Current evidence suggests that resveratrol is cardioprotective in animal models of atherosclerosis, ischemic heart disease, and HF. Though clinical studies for resveratrol have been promising, evidence remains inadequate to introduce it to the clinical setting. In this narrative review, we have comprehensively discussed the relevant compelling evidence regarding the efficacy of resveratrol as a new therapeutic agent for the management of atherosclerosis, MI and HF.

## 1. Introduction

In a key report, the World Health Organization identified cardiovascular disease as one of the foremost challenges to be dealt with in order to improve global health as an estimated 17.9 million people globally are adversely affected annually [1]. Alarmingly, cardiovascular disease is expected to increase substantially due to increases in the rates of metabolic syndrome and obesity. Moreover, a new challenge is arising due to corona virus disease-19 related cardiac complications caused by severe acute respiratory syndrome coronavirus 2 [1,2]. In view of this overwhelming global health care burden, new therapies are essential to further improve the health care for cardiovascular disease. To that end, various avenues have been explored for developing new therapies. Not surprisingly, many frontline medications including cardiovascular drugs have their origin credited to natural sources [3,4]. Recent bio-prospecting approaches identified many food-derived nutraceuticals with potential benefits in cardiovascular disease [5,6]. Nutraceuticals are considered as both standalone therapies as well as adjuvant therapies to tackle various chronic diseases [7]. The plant derived bioactives with lesser side effects may offer superior risk-benefit capability in treating chronic disease as well [7]. One of the most explored plant-derived compounds being considered as a potential treatment for cardiovascular disease is a molecule called resveratrol. Resveratrol has been purported to be behind a phenomenon called the French paradox, which identified that [8,9,10] the French population is apparently characterised with a lower occurrence of cardiovascular disease even with an apparent increased presence of cardiovascular risk factors among them [11]. Over the years, it has been explored extensively for its efficacy in cardiovascular disease [11]. Large number of pre-clinical studies have reported that resveratrol protects against heart disease. A few randomised clinical trials also described resveratrol as a cardioprotective agent. This comprehensive and highly focused review will critically evaluate the current evidence regarding the promising cardioprotective ability of resveratrol and its future translational prospects in ischemic heart disease and HF.

## 2. Methods

A focused PubMed/Scopus search was done to find peer-reviewed articles published in journals in English language till September 2021. Ex vivo, in vivo and randomized, placebo-controlled, double-blinded clinical trials along with meta-analyses and systematic reviews were given more precedence for this review to concentrate on the pertinent available articles only. The searches were done with pre-specified keywords based on the topic of this review such as resveratrol, cardiovascular disease, coronary artery disease, atherosclerosis, ischemia/reperfusion, myocardial infraction, heart failure clinical trial, and randomized, and placebo-controlled, double-blind trial either alone or in combination to maximise the search results. References from the bibliography of previously published peer-reviewed articles were also directly accessed when available in English language. The study designs of the pre-clinical and clinical publications being considered were diligently reviewed before inclusion in the manuscript.

## 3. Resveratrol

### 3.1. Classification and Structure

**Figure 1:**
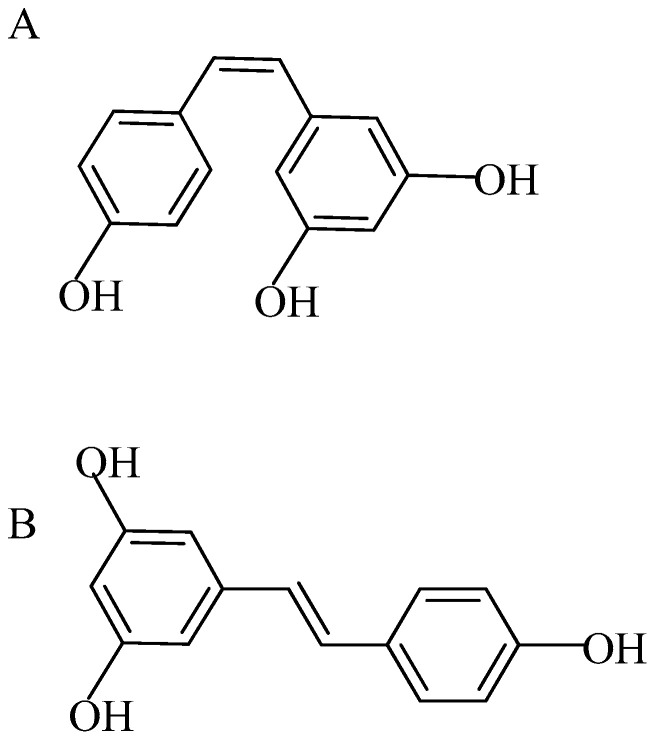
(**A**) Cis-resveratrol; (**B**) trans-resveratrol (molecular weight: 228.24).

Resveratrol falls under a class of molecules known as stilbene polyphenol. Resveratrol is present in many different dietary constituents such as varieties of grapes, berries, plums, and nuts including peanuts in varying amounts [12]. It naturally occurs as small a molecule with dual structural isomeric forms, cis-resveratrol and trans-resveratrol (molecular weight: 228.24 g/mol); they have lipophilic properties, trans-resveratrol being the more biologically active form due to its higher stability (Figure 1A,B) [13].

Phytoalexins form an integral part of the intrinsic immune reaction by plants against infectious diseases [14,15,16]. Plant biologists have established that resveratrol is a phytoalexin that helps plants combat natural stress and infections [12]. An inherent role of resveratrol, possibly attained by natural optimization, as a protective agent appears to have piqued lot of interest in the scientific community. Considering the evolutionary principle that biosynthetic pathways and hormone signalling may have originated from a common evolutionary tree, this natural ability to serve as a disease-modifying agent in plants may be utilised effectively for the management of chronic diseases in humans. For example, salicylic acid in plants offers protection against plant pathogens and blocks jasmonic acid synthesis in plants and its acetylated form aspirin inhibits prostaglandin synthesis in animals through acetylation of prostaglandin synthase/cyclooxygenase. The first line drug therapy candidate for type 2 diabetes is metformin which belongs to a class of compounds called biguanide. It was developed because of the discovery of guanidine in French lilac [17]. It is argued that stress-induced plant compounds may provide protection against stresses in animals as well. However, there are huge challenges in identifying potential bioactive compounds.

Resveratrol is produced in the plants with precursors p-coumaroyl CoA and malonyl CoA in a molar ratio of 1:3. This enzymatic condensation reaction is catalysed by stilbene synthase and protects plants when produced enough [12]. The highest concentration of this polyphenolic compound has been found in the roots of *Polygonum cuspidatum*, a plant which is widely used in oriental traditional medicine. The cardioprotective capacity of resveratrol has been extensively studied after it received a great amount of attention due to its suggested role in “French paradox” [12]. However, a definitive correlation may not be there as several factors could have influenced the “French paradox” such as dietary habits and genetics as well.

### 3.2. Pharmacokinetics of Resveratrol in Humans

The absorption, distribution, metabolism and elimination (ADME) of resveratrol have been extensively studied in humans [18]. The ADME of resveratrol is of great consequence with regards to its ability to replicate in vivo effects demonstrated in in vitro studies. The minimal bioavailability of the free form of ingested resveratrol is often considered as a main obstacle in achieving the beneficial results demonstrated in in vitro studies [18]. That being said, novel resveratrol formulations that can circumvent drawbacks such as limited solubility, and low bioavailability along with a targeted delivery approach may also help to improve its bioavailability and efficacy in the clinical setting.

Interestingly, the oral bioavailability of resveratrol is quite low albeit the total absorption in the intestine is as high as 70%, which is very high compared to other polyphenols [18]. The maximum reported oral bioavailability of resveratrol is only 20% as most of the resveratrol absorbed in the intestine rapidly undergoes conversion to its conjugates in the intestinal epithelial cells and by first-pass metabolism and is eliminated through the kidney. The different metabolites along with trace amounts of unchanged resveratrol namely, 2 resveratrol monoglucuronides, 2 monosulfates, and dihydroresveratrol glucuronide and sulfate were identified after oral and intravenous administration of radio isotope labelled [^14^C] resveratrol. It is also suggested that this bio-transformation phase is a rate limiting factor in terms of resveratrol’s bioavailability as it affects the fate of the absorbed resveratrol in the body. Certainly, the plasma concentration of resveratrol depends on a dose and time dependent regimen and maximum total concentration was estimated as 2.4 µM (~500 ng/mL) after oral administration of a high dose of 5 g whereas a low dose of 25 mg resulted in a very negligible plasma concentration (5 ng/mL) [18,19]. Interestingly, Brown et al. reported an even greater plasma concentration of resveratrol (900 ng/mL) using the same high dose of 5 g in a 29-day study [20]. Of note, the small sample size prevented them from drawing valid conclusions on the safety profile at high dose. Resveratrol half-life was calculated as 1–3 h after single doses and 2–5 h after repeated dosing of 25, 50, 100, and 150 mg and the mean peak plasma levels of 3.89, 7.39, 23.1 and 63.8 nm/L, respectively [21]. Although repeated doses did not result in significantly higher bioavailability, higher doses resulted in higher plasma concentration [21]. Oral ingestion of 5 mg and 1 g [^14^C]-resveratrol resulted in average peak concentrations of 0.6 and 137 μmol/L, respectively.

Resveratrol and its metabolites may also accumulate in different tissues and the latter may bring about their effect in target sites due to the enzymatic breakdown via tissue beta-glucuronidases and sulfatases. In colon cancer patients who have undergone surgery and resveratrol treatment (prior to the procedure), tissue specific distribution of resveratrol metabolites was identified [22]. Additionally, recent studies reported that resveratrol glucuronides and sulfate inhibit colon cancer cell growth [23,24]. It is likely that accumulation of resveratrol in tissues by virtue of its lipophilic nature contributes to some of the beneficial effects observed in various cardiovascular disease models. Brown et al. also reported the toxicity profile from a short duration clinical trial consisting of 44 healthy subjects who consumed resveratrol at daily doses of 0.5, 1.0, 2.5, or 5.0 g [20]. Boocock et al. also reported that high oral doses of resveratrol such as up to 5 g are well tolerated without any serious consequences [19]. The outcome of these studies supports the notion that resveratrol is safe for use by humans as no adverse effects are reported from the biochemical analysis. However, a few gastrointestinal difficulties including diarrhea, flatulence, and stomach ache were observed at the higher doses [20]. These findings were supported by another clinical study involving healthy volunteers also reported no adverse toxicity issues when they underwent treatment with 1 g resveratrol for 4 weeks [25]. Incorporation of metabolic inhibitors of resveratrol biotransformation has been shown to help achieve higher circulating concentration after its administration [26]. Piperine has been shown to inhibit the glucuronidation of resveratrol and improve the pharmacokinetics and slow down its rapid elimination [26]. 

Lastly, novel formulations and a targeted drug delivery system could also improve the bioavailability of resveratrol many folds through improving the solubility and tissue specific delivery. Current formulations used for resveratrol administration suffer from poor aqueous solubility and stabilisation, which affect their pharmacodynamics. Cyclodextrin carrier systems have been found to improve the solubility of resveratrol but it did not significantly augment its bioavailability. Targeted delivery of resveratrol to the desired tissues or increasing the stabilisation of resveratrol in the body by developing sustained release systems can overcome its early degradation in the intestine and liver and its elimination, and thereby augment the bioavailability. Multiparticulate forms and colloidal carriers have been proven as efficient agents for sustained release of resveratrol in specific areas as described elsewhere [27]. Promising outcomes from these innovative approaches to address pharmacokinetic challenges that are associated with resveratrol may help to improve the efficacy of resveratrol in humans.

## 4. Atherosclerotic Cardiovascular Disease, Myocardial Infarction and Heart Failure and Opportunities for Better Management

Coronary artery disease (CAD) impedes the adequate blood flow in the coronaries due to atherosclerosis [28,29]. Atherosclerosis is considered as a slowly developing chronic inflammatory disease of blood vessels that results in the build-up of plaques and is synonymous with CAD/ischemic heart disease as it eventually leads to cardiac ischemia/acute coronary syndrome [28,29]. The risk factors for atherosclerosis include non-modifiable and modifiable factors such as age, sex, family history of CAD, genetic predisposition, cigarette smoking, hyperlipidemia, hypertension, and diabetes mellitus [30]. Endothelial dysfunction that alters vascular homeostasis may precede and pave the way for the initiation of oxidative stress and inflammation and that culminate in formation of atherosclerotic plaques/lesions by a series of pathological changes involving lipid-peroxidation and foam cell formation by macrophages [31]. An atheromatous lesion may be present in the arteries in a clinically dormant stage for a long period of time before it becomes unstable and ruptures or erodes [32]. Neoatherosclerosis and accelerated atherosclerosis are phenomena observed in patients undergoing invasive or surgical procedures for the treatment of CAD and are characterised by rapid development [33]. Atherosclerotic lesion disruption in single or multiple coronary arteries and ensuing thrombosis is the main reason for acute myocardial ischemia also called acute coronary syndrome [30]. Unstable/vulnerable atherosclerotic plaque or thin-cap fibroatheroma rupture causes the activation of platelets due to the exposure of thrombogenic substances to the lumen and contributes to acute decreased blood flow in the coronary circulation [34,35,36]. Even though life style modification and primary and secondary prevention therapies are available to prevent adverse events by reducing risk factors, novel therapies are needed to improve outcomes. This is particularly important considering the fact that none of the current therapies are specifically targeted to reduce oxidative stress and inflammation which contribute to the development and progression of atherosclerosis. 

The human myocardium is not capable of any meaningful large-scale regeneration of cardiomyocytes, to overcome the ischemic cell death and regain its normal functional capacity following ischemia [37]. In contrast, there are a few species of animals which possess an inherent capacity to partially or completely regenerate the myocardium to a functional status after a major injury including newts, teleostean fish, fetal and neonatal rodents [38]. However, the infarcted myocardium of the other species has the ability to orchestrate a wound healing process that effectively replaces the dead cardiomyocytes with a fibrotic scar via a healing process [38,39,40]. The prolonged interruption of the blood supply that precipitates as myocardial ischemia activates a well-described phenomenon of the "wave front" of cardiomyocyte (major contractile muscle cell in the myocardium) death, which originates from the sub-endocardium and radiates through the affected myocardium to the sub-epicardium and results in transmural infarcts [41,42]. Essentially, the post-MI healing encompasses three overlapping stages [38,39,43,44,45]. The inflammatory phase is triggered by endogenous molecules released from necrotic cells via activating pattern recognition receptors including Toll-like receptors [44]. These molecules are capable of activating the innate immune response and are called danger-associated molecular patterns (DAMPs) [45]. Consequently, DAMPs mediated production and release of chemokines and cytokines will result in the recruitment of leukocytes to the infarcted tissue [45]. The recruitment of white blood cells such as neutrophils and macrophages helps to clear dead cells and ECM debris [45]. Neutrophils and macrophages are recruited to the infarct zone of the myocardium by a variety of myocyte-derived factors, including complement C5a, C-X-C motif chemokine 5, monocyte chemotactic protein-1, tumor necrosis factor-α (TNF-α), and interleukins (IL)-1β, IL-6, and IL-8 [45].

The extracellular matrix (ECM) of the myocardium consists of a variety of constituent proteins which can serve as structural and adhesion agents in dynamic contractile machinery that hold together all other components. The scar development progresses by the process of cross-links formation of collagen fibers to attain sufficient tensile strength to withhold the force generation and to partially maintain the normal geometry of the left ventricle (LV) to ensure that loss of functional tissue does not severely limit cardiac function [39]. Adequate collagen deposition in the scar tissue is pivotal to retain the tensile strength and prevent a rupture of the infarcted LV wall due to the loss of viable tissue [46]. The post-MI scar formation and maturation continues for a few weeks and this well-regulated process culminates with a fully developed scar at around eight weeks [34,40,46]. In addition, the increased workload on non-infarcted LV and the resultant elevated mechanical stress also contributes to the activation of transforming growth factor-β 1 (TGF-β 1) within the myocardium which is a potent pro-fibrogenic molecule [47,48]. Cardiac fibroblasts are distributed throughout the heart between cardiac muscle fibers [39,46]. Increased renin-angiotensin-aldosterone system (RAAS) activity and the release of TGF-β 1 are known to cause the activation of cardiac fibroblasts into myofibroblasts that will result in cardiac fibrosis [47,48]. The healing of the infarcted LV tissue forms an indispensable process that is coupled with the geometric remodeling of the LV chamber. The necessary evil of scar formation has been shown to predispose the myocardium to a trajectory of progressive LV wall thinning, and dilatation and LV systolic and diastolic dysfunction [47,48]. Targeting cardiac modeling is generally perceived as a critical therapeutic window of opportunity to prevent the development and progression of HF [49]. Currently there are no specific drug therapies for to limit MI induced cell death or injury to the myocardium. Moreover, our ability to intervene the process of post-MI remodeling and cardiac fibrosis has also been limited.

Heart failure (HF) is a clinical condition regarded as the inability of the myocardium to sustain sufficient organ perfusion in the body due to inefficient myocardial filling and pumping capacity secondary to the damage and/or weakening of the myocardium [50,51,52]. HF is a multifactorial life threatening and debilitating syndrome that is identified clinically with a diagnosis process ranging from careful analysis of patient history, physical examination, non-invasive cardiac imaging and biochemical tests, and is targeted with a multifaceted treatment regimen [50,53]. As per the most recent guidelines, HF is classified into three main types based on different thresholds of LV functional status in patients represented by LV ejection fraction (LVEF) [53]. HF patients with normal LVEF (which is quantified as ≥50%) comes under the category of HF with preserved LVEF (HFpEF) and patients with reduced LVEF (usually <40%) belong to the category of HF with reduced EF (HFrEF) [53,54]. Patients that have LVEF of 40–49% are considered as another category and they are classified as HF with midrange EF (HFmrEF) [54]. Even with the highly optimized current therapies the burden of HF remains enormous.

## 5. Evidence of Resveratrol Mediated Protection against Atherosclerotic Cardiovascular Disease

Resveratrol is considered as a molecule of interest in managing the risk factors of atherosclerosis and its development and progression due to its multi-faceted action. In this regard, resveratrol has been shown to lower atherosclerosis risk factors such as total cholesterol (TC), low density lipoprotein cholesterol (LDLC), very low-density lipoprotein cholesterol (VLDLC), apolipoprotein-B (Apo B), lipoprotein-A (Apo A), free-fatty acids (FFA) and triglycerides (TG) [55,56,57]. It also increases the level of “good cholesterol” such as high-density lipoprotein cholesterol (HDLC) [55,56,57]. Resveratrol may also reduce oxidized-LDLC due to its antioxidant action. Interestingly, treatment with resveratrol also reduced the progression of fatty streak formation in ApoE (^−/−^) mice under a standard diet suggesting its anti-atherogenicity potential [58]. Resveratrol also prevented higher level of TC, TG, LDLC and HDLC and reduced the atherosclerotic lesion induced by high fat diet and lipopolysaccharide further confirming its lipid lowering and anti-atherosclerosis potential [59]. In another mouse model of human cardiovascular disease, APOE*3-Leiden.CETP, resveratrol reduced atherosclerosis lesion and improved plaque stability as well comparable to cholesterol lowering drug atorvastatin in mice [60]. On the other hand, resveratrol and atorvastatin combination was not superior in reducing atherosclerosis even though there was a slightly increased benefit [60]. In contrast, resveratrol acted synergistically with pravastatin in rat fed 2% high cholesterol diet to improve the lipid profile [56]. Resveratrol may mediate its action via an enzyme in the liver called 3-hydroxy 3-methylglutaryl coenzyme A (HMG-CoA) reductase. Resveratrol is known to modulate the expression of HMG-CoA reductase in hamsters [61]. Resveratrol also increased the expression of cholesterol 7α-hydroxylase in liver associated with bile acid production which can lower TC and LDLC. Overall, resveratrol appears to be a suitable candidate for lowering the risk of atherosclerosis. 

**Figure 2:**
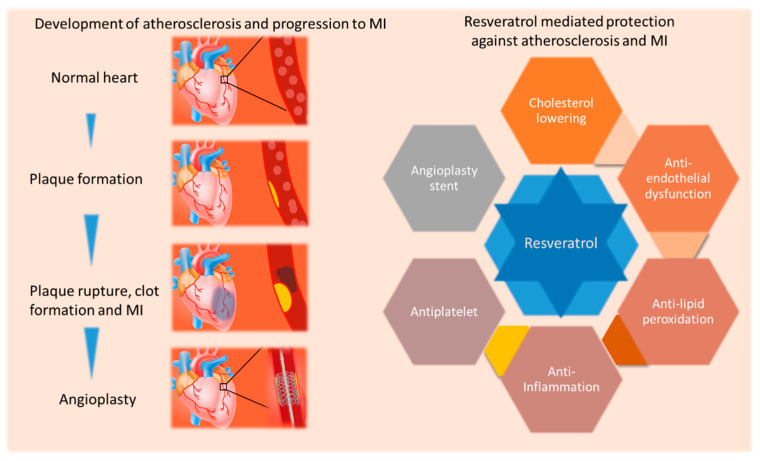
Schematic depicting resveratrol mediated reduction in ischemic heart disease by targeting various factors. Atherosclerosis develops due to lipid accumulation and inflammation in arteries leading to the plaque formation, plaque rupture, thrombus formation and MI. Resveratrol decreases the level of bad cholesterol, lipid peroxidation, inflammation and platelet aggregation and improves endothelial function and re-endothelialization (when use in drug-eluting stents), suggesting manifold action of resveratrol in improving ischemic heart disease.

However, resveratrol does not show a be-all and end-all lipid lowering effect to produce anti-atherosclerosis action. It is interesting to note that it may also produce anti-atherosclerosis outcome in the absence of a lipid lowering effect. This signifies the role of resveratrol in modulating the pathogenesis of atherosclerosis via other signaling pathways (Figure 2). Notably, resveratrol also reduced trimethylamine-N-oxide (TMAO)-induced atherosclerosis in ApoE^−/−^ mice by decreasing TMAO levels via reducing trimethylamine (TMA) by altering gut microbiota in mice [62]. Resveratrol also reduces intestinal fatty acid and monoglyceride in male ApoE^−/−^ mice [63]. In ApoE^−/−^Fas^−/−^ double knockout mice, which display a lupus profile with accelerated atherosclerosis, resveratrol was able to prevent atherosclerosis progression [64]. In higher species such as rabbits also resveratrol was able to produce favorable effects. For example, resveratrol treatment reduced the levels of TC, LDLC, lipoprotein-associated phospholipase A2, and creatinine in rabbits fed a high fat diet [65]. In rabbits fed a hypercholesterolemic diet, dealcoholized red wine and resveratrol both lowered the size, density, and atherosclerotic plaques, and thickness of the intima layer and improved flow-mediated dilation without changing lipid profile [66]. Hence it is possible that resveratrol reduces the risk of atherosclerosis even when they do not affect lipid levels. Resveratrol dietary supplementation also prevented central arterial wall stiffening and inflammation in non-human primates on a chronic diet high in fat and sucrose suggesting the efficacy of resveratrol in higher species close to humans against atherosclerosis risk factors [67]. However, a meta-analysis of randomized controlled trials showed that resveratrol does not affect TC, LDLC, triglycerides and HDLC [68]. That being said, large, randomised trials are need to be conducted in high-risk population to understand the role of resveratrol in providing benefits as current lipid lowering therapies. Resveratrol may also be explored for its efficacy in primary prevention of major cardiovascular events in high-risk patients.

Platelet aggregation plays an important role in acute coronary syndrome and resveratrol appears to block this process [69,70]. Endothelium is known to maintain a dynamic balance between vasodilators and vasoconstrictors. Resveratrol has been reported to positively influence and maintain vascular homeostasis. Resveratrol promotes vascular homeostasis via vasodilation and inhibiting monocyte adhesion and vascular smooth muscle proliferation through endothelial nitric oxide synthase (eNOS). Hence, resveratrol may be effective as an antiplatelet agent as well.

## 6. Novel Reperfusion Therapy Approaches with Resveratrol

Angioplasty with drug eluting stents is a preferred treatment for stenosis and occlusions in coronary arteries associated with MI. However, there are unavoidable drawbacks with this therapy as well. Interestingly, a rodent study mimicking arterial angioplasty and stenting with resveratrol-eluting stents tested the effects resveratrol on neointimal hyperplasia and re-endothelialization [71]. The delivery of resveratrol from a drug-eluting stent reduced in-stent stenosis, while promoting re-endothelialization [71]. This may be helpful in lowering the risk of late-thrombosis seen with drug-eluting stents. Another study investigated the effects of angioplasty with resveratrol-coated balloon catheters in coronary and peripheral arteries [72]. A reduction in the number of microvessels and macrophages in the adventitia and improvement in re-endothelialization were also happened in peripheral arteries with treatment [72]. In addition, paclitaxel/resveratrol-coated balloon catheter was tested for the transport of the coating and subsequent beneficial effects [73]. Specifically, inhibition of neointimal proliferation, inflammation and tolerance of complete coating and resveratrol-only coating was investigated in pigs for four weeks after treatment [73]. In peripheral arteries with resveratrol-only balloons, inflammation and fibrin deposition were lower along with a reduction in macrophage and a pronounced re-endothelialization [73]. Harmful complications were not noticed with high-dose treatment of coronary arteries [73]. In adult male New Zealand White rabbits, intravascular administration of resveratrol by drug-delivery catheter after the induction of intimal hyperplasia by traumatic angioplasty resulted in an inhibition of intimal proliferation [74].

## 7. Resveratrol as a Treatment for MI and HF

### 7.1. Evidence of Resveratrol Mediated Protection in Myocardial Ischemia/Reperfusion Ex Vivo

Resveratrol has been extensively explored for its cardioprotective action against myocardial ischemia/reperfusion injury. Initial ex vivo studies specifically focused on the potential of resveratrol as an ischemic preconditioning agent as it has been an area of avid interest (Table 1). Resveratrol pre-treatment reduced infarct size in explanted ischemic reperfused hearts (an ex vivo model of MI) suggesting its protective action as an ischemic preconditioning molecule with a potential mechanism of action involving glycogen synthase kinase-3-beta and mitochondrial permeability transition pore [75] Pre-treatment with resveratrol before ischemia and reperfusion led to improved LV function and infarct size [76]. Resveratrol also reduced tachyarrhythmia in left anterior descending artery (LAD) ligated and reperfused rat hearts [77]. Resveratrol also caused an improvement cardiac function following global myocardial ischemia and reperfusion and reduced single and salvo arrhythmias, ventricular tachycardia, ventricular fibrillation, infarct size, creatinine kinase-MB, lactate dehydrogenase, and troponin I [78]. Ten minutes resveratrol treatment was also able to limit infarct size after regional ischemic episode and reperfusion in male Wistar rats [79]. Resveratrol pre-treatment of TNFR2 wild-type mice heart subjected to an ischemia–reperfusion insult also reduced infarct size [75]. However, in one study, pre-treatment with resveratrol in isolated rat hearts under ischemia and reperfusion did not alter the functional parameters [79].

Longer duration of treatment with resveratrol before ischemia/reperfusion has been proven beneficial in relieving myocardial ischemia/reperfusion injury ex vivo. Resveratrol has also been able to preserve cardiac function when the heart was subjected ischemia/reperfusion ex vivo [80]. One-week prior treatment with resveratrol at varying doses also recovered the post-ischemic cardiac function ex vivo and improvement was correlated with the doses [81]. The hearts from rats which received resveratrol for fifteen days showed better cardiac function after 60 min low-flow ischemia/reperfusion [82]. Resveratrol also improved CF and LV pressure, and reduced infarct size, and apoptosis in the hearts after ischemia/reperfusion injury [83]. Lengthy pre-treatment with resveratrol (16 weeks) also reduced infarct size after ischemia/reperfusion ex vivo [84]. Resveratrol has also been shown to offer cardioprotection in ischemia/reperfusion injury in animals with co-morbidities in ex vivo studies. Specifically, a study showed that pre-treatment with resveratrol for two weeks reduced VF and infarct size as well as improved LV pressure, CF and aortic flow in 10% glucose treated or non-treated Zucker obese rats after ischemia and reperfusion [85]. Furthermore, it also improved LV developed pressure, and led to a limited area of infarcted tissue when the hearts were under ischemia and reperfusion in streptozotocin-induced diabetic rats [86]. Resveratrol pre-treatment for two weeks also led to better LV developed pressure and reduced infarct size after ischemia and reperfusion ex vivo in rats which received a hypercholesteraemic diet [56]. Specifically, two weeks of pre-treatment with resveratrol improved peak systolic pressure, +dP/dt max, and CF diastolic pressure and infarct size [56].

A meta-analysis also showed that pre-treatment with resveratrol was also associated with a significant limitation in infarct size after ischemia–reperfusion insult ex vivo in mice and rats without any co-morbidities [87]. Based on the studies reviewed above, resveratrol treatment of varying duration prior to induction of ischemia and reperfusion ex vivo may offer pre-conditioning benefits. It is not known whether resveratrol is more effective when the myocardium is under ischemia or during reperfusion, i.e., at what point resveratrol provide benefits as ischemia and reperfusion may differently affect myocardium is still not known. MI increases arrhythmogenesis and causes sudden cardiac death due to large infarcts and causes cardiac remodeling. Reduction of infarct size after ischemia/reperfusion is important as it will in turn reduce arrhythmogenesis and cardiac remodeling. Resveratrol may be explored for its benefits in reducing the damage due to reperfusion therapy involving thrombolytic drugs, percutaneous coronary intervention, and no-reflow phenomenon that occurs during primary percutaneous intervention. Resveratrol may be effective in reducing ischemia/reperfusion injury when a pre-existing condition is present [56,86]. 

### 7.2. Effects of Standalone and Combination Resveratrol Treatment on Ischemia and Ischemia/Reperfusion, Permanent Ischemia, and HF In Vivo

Short-term administration of resveratrol also provides cardioprotection against ischemia and reperfusion in vivo. Pre-treatment with resveratrol before induction of ischemia by LAD ligation followed by reperfusion improves LV pressure and reduces infarct size in rats [88]. Resveratrol also recovers cardiac function and reduces infarct size and myocardial apoptosis in vivo, when given five minutes before ischemia/reperfusion [89,90]. Resveratrol is reported to decrease ischemia/reperfusion-induced ventricular VT and VF and reduce infarct size [91]. Resveratrol pre-treatment also reduced VT and VF only after left main coronary artery occlusion/reperfusion in rats, not after left main coronary artery occlusion alone [92]. However, in LAD ligated rats without reperfusion, resveratrol reduced arrhythmia, VT and mortality in a dose dependant manner [93]. Resveratrol treatment 24 h before 30 min occlusion of the LAD and 24 h reperfusion decreased infarct size in mice [94]. Resveratrol treatment also improved cardiac hemodynamics decreased mortality, infarction area, fibrosis, and cell apoptosis [95]. It also reduced the expression of markers of senescence (p53, p16, and p19), inflammasome and Cas1 p20, interleukin- (IL-) 1β, IL-6, TNF-α, and IL-18 and translocation of NF-κB to nucleus in vivo [96]. Resveratrol treatment also reduced apoptosis and improved cardiac function after myocardial ischemia/reperfusion in male mice [97]. Interestingly, short-term resveratrol was able to provide benefits in the presence of co-morbidities and MI in vivo as well. As mentioned above, resveratrol also mitigated the ischemia reperfusion induced myocardial damage and infarct size and improved hemodynamic function in streptozotocin-induced diabetic rats in vivo [98].

It is worthwhile to explore the long-term benefits of resveratrol treatment in vivo, especially MI related remodeling that can lead to cardiac dysfunction and HF. Resveratrol pre-treatment for two weeks reduced infarct size and improved cardiac function, perfused capillary network and myocardial blood flow, in 24 h LAD occluded rats under normal state and dobutamine stress condition [99]. Resveratrol pre-treatment began 1 week before LAD ligation and carried out for next three weeks reduced infarct size and LV hypertrophy, and blocked VT and VF and inducible VT [100]. Survival rate was also better in MI-induced rats treated with resveratrol [100]. Resveratrol pre-treatment (1 week) also reduced infarct and improved capillary network and the first derivative of LV developed pressure three after LAD ligation [101]. Its administration in MI-induced rabbits improved LV function, and reduced atrial remodeling and atrial fibrosis, which also led to protection from atrial fibrillation (AF). Protection against AF was attributed to a positive modulation of eNOS [102]. It also improved LV function, and decreased interstitial fibrosis, cardiac hypertrophy, and the level of plasma brain natriuretic peptide (BNP) induced after isoproterenol (ISO) treatment induced MI [103]. Cardiac function and mortality have been shown to be improved with resveratrol treatment in MI-induced mice by promoting the transition of macrophages towards the M2 phenotype [104]. Resveratrol supplementation also decreased the inflammatory cytokine levels, cardiac dysfunction, and atrial interstitial fibrosis in MI-induced rats [105].

Treatment with resveratrol for four weeks after induction of an MI also improved infarct size and LV structure and function in rats [106]. It was also able to reduce infarct size, and cardiac remodeling, and produce improved LV function and survival rate [107]. Resveratrol treatment at a low dosage for sixteen weeks also reported an improved cardiac structure and function, and an increased survival rate in MI-induced rats [108]. Resveratrol has been shown to reverse established ischemic HF. Resveratrol treatment for two weeks, began after four weeks after LAD ligation in mice, also resulted in an improvement in cardiac remodeling and cardiac function [109]. MI-induced mice had developed HF (LVEF < 40%) prior to resveratrol treatment and treatment was able to induce reverse cardiac remodeling HF and improve LV dysfunction [109]. Recently, another pre-clinical study reported the efficacy of resveratrol in improving systolic function in the setting of post-MI related HF [110], which showed that three weeks after LAD-ligation, MI rats had signs of HF (LVEF < 40%), and low-dose resveratrol treatment for 2 weeks (started 3 weeks post-surgery and HF was established) improved LVEF. After LAD ligation, repeated echocardiography showed very long-term (10 months) resveratrol treatment via diet reduced pulse wave velocity and LV dysfunction [111]. Interestingly, we also observed that resveratrol protects from cardiac remodeling and dysfunction in male and female rats with female MI rats showing a better response [112]. However, more studies are needed to understand the sex differences in the efficacy of resveratrol in MI related complications.

Resveratrol pre-treatment for four weeks in Yorkshire swine on a hypercholesterolemia diet followed by another three more weeks after left circumflex artery occlusion conserved local wall motion, improved flow during LV pacing, and increased vasodilation in the ischemic heart [113]. Resveratrol also lowered cholesterol levels in hypercholesterolemia swine [113]. Pre-conditioning and post-conditioning by resveratrol has been attributed to beneficial effects in the ischemic myocardium. Pre-supplementation with resveratrol lead to improved LV function and perfusion to the non-ischemic zone in a similar model of metabolic syndrome and chronic myocardial ischemia mentioned above [114].

Contrary to the studies discussed above, some studies have also reported that resveratrol treatment was not effective in MI-induced animals. For example, in male New Zealand white rabbits, resveratrol did not affect infarct size or improve regional myocardial blood flow after MI [115]. In another study, thirteen weeks combined pre- and post-treatment with resveratrol after MI did not improve cardiac function [116], although the cardiac performance was better with the dobutamine stress test in MI-induced rats suggesting partially beneficial effects [116]. Four weeks and two weeks resveratrol treatment in permanent LAD ligated rats and mice also did not offer cardioprotection with lower doses [106,109]. In the reported studies, administration of resveratrol has been done via different routes such as gavage, intraperitoneal injection and via diet. In addition, animal strains, vehicles (ethanol, dimethyl sulfoxide (DMSO), and water), variation in LAD ligation and doses used by various studies were also different. These discrepancies may have contributed to the variability in the outcomes of the studies. 

In order to be successful in a clinical setting, resveratrol treatment should provide beneficial patient outcomes in ischemic heart disease patients being treated with existing medications. Only a few studies have been carried out to unravel the potential of resveratrol as a combination therapy together with proven cardiac medication [56]. A combination of resveratrol and hydralazine decreased myocardial ischemia/reperfusion injury ex vivo in wild type and eNOS deficient mice [84]. In another study in hypercholesterolemic diet fed rats, a combination of resveratrol and statin resulted in an increase in LV function and smaller infarct size compared to ischemia-reperfused hearts from a hypercholesterolemic diet fed rats that received either of the interventions alone [56]. The combination of statin and resveratrol may also improve outcomes after ischemia or ischemia/reperfusion injury in vivo. The efficacy of the combination treatment has to be established in MI setting in vivo as well. In this regard, our study showed that low dose resveratrol was as effective as an ACE inhibitor, perindopril, in preventing cardiac remodeling and dysfunction in MI-induced young rats [117]. Evidence reveals that both pre-treatment or post-treatment with resveratrol in ischemia and ischemia/reperfusion, permanent ischemia and ischemic HF are effective in small and large animal models in vivo (Table 2).

### 7.3. Role of Resveratrol in microRNA Therapeutics, Nanocrystal Self-Assembled Microspheres, and Enhancing Cell Therapy and Tissue Grafts

MicroRNAs (miRs) have been described as smaller sized non-coding RNAs regulators of various molecular events [118]. Resveratrol has been explored for its role in modifying miRs under pathological conditions [119,120]. Resveratrol was able to bring about alterations in the miR levels after ischemia/reperfusion ex vivo. These changes lead to improvement in cardiac function and a reduction in infarct size [120,121]. Resveratrol altered miR expression in ischemic heart disease patients [122]. Further studies are needed to reveal the efficacy of miRs in the in vivo settings. Resveratrol was also able to alter the expression of microRNA-34a in ISO-challenged male Sprague–Dawley rats [123]. Further studies are needed understand how resveratrol affects the miR expression. As there are various emerging miR based approaches, it will also be worthwhile to investigate a combination treatment with resveratrol and other miR based therapies to as well.

Orally administrated treatments have to escape the degradation of active forms in the gut, which is a main obstacle for bioavailability [124]. Nanoparticles are used to increase the favourable bioavailability of administrated treatments. Spray dried nanocrystals are capable of forming aggregates and it can improve desired properties for better bioavailability [124]. Nicotinamide riboside (NR) is a pyridine nucleoside form of vitamin B3. Resveratrol and NR nanocrystal self-assembled microspheres reduced infarct size and improved LVEF after cardiac ischemia/reperfusion injury in mice [124]. Pre-treatment with poly(lactic-co-glycolic (PLGA) acid nanoparticle loaded with resveratrol prevented myocardial necrosis and reduced interstitial edema and neutrophil infiltration ISO induced MI in rats [125]. PLGA is a United States Food and Drug Administration (USFDA) approved biodegradable polymer. Mitochondria-targeted nanoparticle mediated delivery of resveratrol increased its distribution in the ischemic myocardium and reduced infarct size and cardiac apoptosis while maintaining the integrity of mitochondria [126]. Methoxy poly(ethylene glycol)-*b*-oligomerization(D, L-Leucine) (mPEG-*b*-O(D, L-Leu)) nanoparticle carrier containing resveratrol protective against ischemia/reperfusion [127].

Inducing myocardial cell therapy may help in managing MI related cardiac complications [128,129]. Regenerative therapy has progressed from a novel concept to translational stages over the years with many large pre-clinical studies and ongoing clinical studies [128,129]. Cell therapy has many unanswered challenges limiting its efficacy including impaired engraftment retentiveness and persistence of stem cells [129]. Resveratrol may enhance mobilisation of stem cells in the injured heart [130]. Autologous Sca-1^+^Lin^−^CD45^−^CXCR^+^ very small embryonic-like stem cell (VSEL) mobilisation may provide cardioprotection [130]. Resveratrol resulted in increased recruitment of VSEL to the injured myocardium compared to statin [130]. Cardiac stem cells (CSC) from the explanted decompensated hearts (E-CSC) are potential candidates MI induced damage. They suffer from the drawback of cellular senescence, which reduces their regenerative capacity. Ex vivo pre-treatment with resveratrol and rapamycin has been shown to reduce senescence of E-CSC, which may help in circumventing this complication. Transplantation pre-treated E-CSC also reduced cardiomyocyte senescence and apoptosis and increased endogenous c-Kit^+^ CSC in the peri-infarct zone. This revealed that resveratrol improves the viability of stem cells [131]. Resveratrol administration causes cardiac stromal cell-derived factor (SDF)-1 upregulation and can enhance the mobilization of stem cells in mice with acute MI [95]. Cardiac progenitor cells treated with resveratrol also improved the survival of mice via the mitochondrial activation in myocardium [55]. Resveratrol activated endogenous stem cell antigen-1-positive (Sca-1^+^) cardiac stem cells also increased capillary network and reduced apoptosis in the peri-ischemic area, and enhanced the effects of CSCs transplantation [132]. Transplantation of resveratrol treated bone marrow mesenchymal stem cells to MI induced diabetic rat was also beneficial [133]. Oxidative stress, increased pro-inflammatory response, lack of blood supply and apoptosis may affect differentiation of stem cells in the myocardium [129]. Resveratrol appears to improve cardiac regenerative therapy and hence its efficacy in enhancing regenerative therapy may be further explored. 

Tissue-engineered grafts and scaffolds may be useful to provide support to the myocardium after an MI. Direct transfer of resveratrol to the injured heart muscle is also an area of interest due its high efficacy but low plasma bioavailability of the native form after oral administration. Resveratrol (5 mg/mL) and ferulic acid loaded in core–shell electrospun fibers made of chitosan and polycaprolactone (PCL) graft on full-thickness excision skin wounds has been shown to result in 100% wound closure by two weeks [134]. Resveratrol loaded PCL fibrous scaffold also leads to cellular migration, nitric oxide production, rapid vascularization and endothelialization of small diameter vascular graft [135]. Embedded resveratrol in a polycaprolactone scaffold also reduced inflammatory cell infiltration and better collagen extracellular matrix secretion and blood vessel network formation in MI-induced mice [136]. It is apparent that there.

### 7.4. Resveratrol Mediated Cardiac Protection in Ischemic Heart Disease and HF Patients

**Figure 3:**
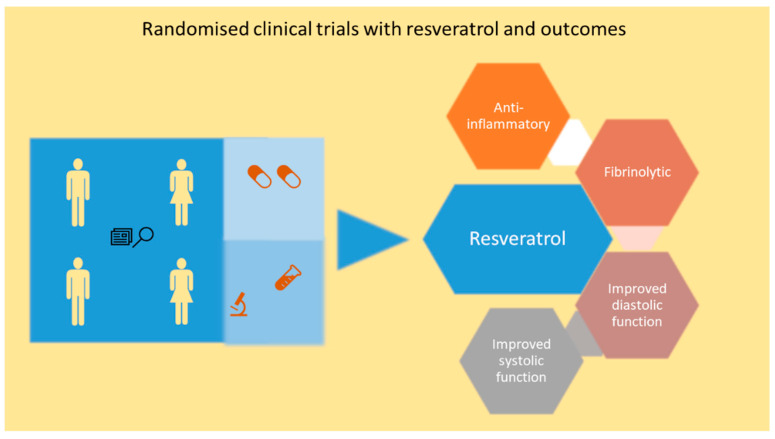
Schematic depicting the resveratrol mediated improvement in clinical parameters in randomized clinical trial in CAD and HF patients. Resveratrol reduces pro-inflammatory markers and induces fibrinolysis in CAD patients. Resveratrol also improves diastolic and systolic function in CAD and HF patients.

Due to the evidence from a large number of pre-clinical studies, a great level of enthusiasm exists for exploring the cardioprotective role of resveratrol in patients with ischemic heart disease and HF. In this section, evidence from clinical trials will be analyzed (Table 3 and Figure 3). To date, pure resveratrol and resveratrol-enriched grape extract have been used in clinical trials suggesting diverse avenues of clinical strategies which can be pursued with resveratrol.

A double-blinded, placebo-controlled, randomized, three-month clinical study showed that resveratrol treatment in 40 post-MI patients including 26 men and 14 women was able to improve diastolic function and systolic function. The study consisted of placebo and 10 mg/day resveratrol group, and both groups also received routine post-MI medication. Endothelial function, red blood cell deformability, LDLC and platelet aggregation were also improved in resveratrol treated group [137]. TC, HDLC, TG and high-sensitivity C-reactive protein (h-CRP) were not affected with resveratrol treatment. Calcium fructoborate (CF) is a dietary supplement that has calcium, fructose, and boron in a sugar-borate ester as the three main ingredients with anti-inflammatory action [138]. A clinical trial also studied the efficacy of three-month treatment with resveratrol and CF in 116 patients with stable angina pectoris [139]. Twenty-nine patients in three groups received placebo treatment, or 20 mg/day resveratrol alone, or a combination of 20 mg/day resveratrol and 112 mg/day CF, or 112 mg/day CF. Treatment led to reductions in h-CRP and the N-terminal pro-hormone of BNP in all three groups. LDLC and TG were also lower in all the treatment groups. CF and resveratrol treatment led to lower incidence of angina episodes, and the number of weekly nitroglycerin consumption, and better angina class. Due to the relatively shorter duration of the trial, it was not possible to ascertain long-term effects of resveratrol treatment. Another randomized trial with 85 CAD patients also reported the effects of resveratrol treatment at a dose of 100 mg per daily (added to standard treatment) for two months. CAD patients treated with resveratrol had improved diastolic function and systolic function [140]. A randomized, triple-blinded, placebo-controlled trial with 75 stable angina patients who received either a regular grape extract or a grape extract containing resveratrol for one year showed benefits such as reduction in pro-inflammatory biomarkers and improved anti-inflammatory and fibrinolytic biomarkers [141]. The grape extract (350 mg/day) containing 8 mg resveratrol was given in the initial 6 months and followed by grape extract (700 mg/day) containing 16 mg resveratrol for next 6 months of the study. Patients also received standard medication. Resveratrol treatment increased adiponectin and decreased plasminogen activator inhibitor type 1 [141]. It also resulted in a down-regulation of pro-inflammatory genes and changes in transcription factors and miRs in two subpopulations of patients in the trial [142]. The lipid profiles of the patients were also improved with treatment [142]. The first two studies are particularly interesting due to the fact that the concentration of resveratrol treatment was lower. Both studies support the fact that resveratrol in low dose may also be beneficial. 

In a HF clinical trial (Table 3), 60 out patients with NYHA (New York Heart Association) class II to III HFrEF were randomized to receive either resveratrol 100 mg/day or placebo for three months and cardiac ultrasound, six-minute walk test, quality of life questionnaire, spirometry and RNA analysis were done. Resveratrol treated group showed better LV function and global longitudinal strain than placebo group [143]. A sub-study of this trial also showed that resveratrol treatment had no effect on hematocrit and viscosity. The erythrocyte deformability also was unaffected whereas, there was an improvement in red blood cell aggregation within resveratrol group at three months vs. baseline. Resveratrol also resulted in better exercise tolerance and it could be attributed to better cardiac function. A positive correlation also existed between the exercise capacity and the hemorheological properties. It should be noted that number of patients and duration of the study are lower compared to landmark HF trials.

**Table 3:** Effects of resveratrol in improving various clinical parameters in randomized clinical trials in stable CAD patients.

Clinical Study Design	Duration of the Study and Dose of Resveratrol	Main Clinical Outcomes
Randomized, double-blinded, placebo-controlled, 40 post-MI patients	3-month, 10 mg/day resveratrol	Improved endothelial function, red blood cell deformability, LDLC and platelet aggregation [137]
Randomized, double-blinded, placebo-controlled, 116 patients with stable angina pectoris	2-month, 20 mg/day resveratrol	Lower incidence of angina episodes, and the number of weekly nitroglycerin consumption, and better angina class [139]
Randomized trial with 85 CAD patients	2-month, 100 mg/day resveratrol	Improved diastolic function and systolic function [140]
Randomized, triple-blinded, placebo-controlled trial with 75 stable angina patients	6-month grape, 8 mg/day resveratrol and 16 mg resveratrol for next 6 months	Increased adiponectin and decreased plasminogen activator inhibitor type 1 and pro-inflammatory gene expression [141,142]
Randomized, double-blinded, placebo-controlled trial with 60 out patients with NYHA class II to III HFrEF	3-month 100 mg/day resveratrol	Better LV function and global longitudinal strain [143]

In view of the emerging clinical trial data, large randomized clinical trials should be conducted in relevant patient population such as persons with risk of CVD, and post-MI and HF patients. This will lead to development of resveratrol as novel therapy for managing cardiovascular disease.

## 8. Conclusions

This comprehensive review concludes that current evidence from pre-clinical studies strongly support the role of resveratrol as a very promising bioactive molecule with a wide range of cardioprotective action in the context of ischemic heart disease and HF. In essence, the emerging clinical evidence also largely corroborate this observation. Further clinical studies are needed to fulfil the promise of the translational potential of resveratrol in ischemic heart disease and HF.

## Figures and Tables

**Table 1:** Ex vivo evidence of resveratrol mediated cardioprotection in ischemia and ischemia/reperfusion.

Duration and Dose of Resveratrol Treatments	Ischemia or Ischemia/Reperfusion Durations	Outcomes
Five minutes prior to the onset of ischemia 10 μM	Thirty minutes Ischemia and 120 min reperfusion	Reduced infarct size [75]
Fifteen minutes prior to the start of ischemia (20 and 100 μM)	Twenty minutes ischemia and 30 min reperfusion	Reduced infarct size and improved cardiac function [76]
Before ischemia 2.3 mg/L	Thirty minutes Ischemia and 45 min reperfusion	Reduced in infarct size [77]
Seven days treatment with 25 mg/kg body weight/day	Forty-five minutes ischemia and 10 min reperfusion	Improved in cardiac function [80]
Seven days 2.5, 10, 25, and 50 mg/kg body weight/day	Fifteen minutes ischemia and reperfusion 10 min	Improved cardiac function [81]
Fifteen days 25 mg/L	Fifteen minutes ischemia and 10 min reperfusion	Improved recovery of cardiac function and vasodilation [82]
Six weeks of 2 mg/kg body weight/day	Thirty minutes ischemia and 30 min reperfusion	Improved LV pressure, CF and reduced infarct size [83]
Sixteen weeks of 25 mg/kg body weight/day in diet	Fifteen minutes ischemia and 30 min reperfusion	Reduced in infarct size [84]
Two weeks of 5 mg/kg body weight /day	Thirty minutes ischemia and 120 min reperfusion in Zucker obese rats	Improved cardiac function [85]
Fifteen days 2.5 mg/kg body weight/day for	Thirty minutes ischemia and 120 min reperfusion in streptozotocin-induced diabetic rats	Improved LV pressure, and reduced infarct size [86]
Two weeks with 20 mg/kg body weight/day	Thirty minutes ischemia and 120 min reperfusion in rats on hypercholesterolemic diet	Improved LV pressure and reduced infarct size [56]

**Table 2:** In vivo evidence of resveratrol mediated cardioprotection in ischemia and ischemia/reperfusion.

Duration and Dose of Resveratrol Treatments	Ischemia or Ischemia/Reperfusion Durations	Outcomes
Fifteen minutes 10 μM before induction of ischemia	Thirty minutes ischemia by LAD ligation and 120 min reperfusion	Decreased LV systolic pressure and reduced infarct size [88]
Five minutes prior to reperfusion 100 μM/L	Thirty minutes ischemia by LAD ligation and 120 min reperfusion	Reduced infarct size [89]
Sixty minutes prior to induction of ischemia	LAD ligation and subsequent reperfusion	Reduced infarct size and decreased VT and VF [91]
1 μM	Five or 30 min LAD ligation and 30 min reperfusion	Lower incidence and duration of VT and VF [92]
Ten minutes prior to the surgery 5, 15, and 45 mg/kg body weight	LAD ligation	Lower duration of arrhythmia and decreased VT and mortality [93]
Twenty-four hour prior to 30 min (intraperitoneal administration—5 mg/kg body weight)	LAD ligation	Reduced infarct size [94]
Two weeks 1 mg/kg body weight/day	LAD ligation	Reduced infarct size and improved cardiac function [99]
One week prior to LAD ligation and an additional 3 weeks 5 mg/kg body weight/day	LAD ligation	Reduced infarct size and cardiac hypertrophy, and VT and VF [100]
One week 10 mg/kg body weight/day	LAD ligation	Reduced infarct size. Improved capillary density and LV developed pressure [101]
Four weeks as pre-treatment and post-treatment for another 3 more weeks 100 mg/kg body weight/day	Induction of left circumflex artery constriction	Preserved regional wall motion, better flow augmentation with ventricular pacing, and increased vasodilation [113]
Four weeks as pre-treatment and post-treatment for another 3 more weeks 100 mg/kg body weight/day	Induction of left circumflex artery constriction	Improved regional LV function and preservation of perfusion [114]
Four weeks by IP injection 1 mg/kg body weight	LAD ligation	Improved LV dilatation, systolic and diastolic function and reduced infarct size [106]
Six weeks 20 mg/kg body weight/day	LAD ligation	Decreased infarct size, and cardiac remodeling, and improved LV function and increased survival rate [107]
Eight weeks and sixteen weeks 2.5 mg/kg body weight/day	LAD ligation	Improved cardiac structure and function, and survival [108,117]
Two weeks osmotic pump treatment (started 4 weeks after ligation—50 mg/kg body weight/day)	LAD ligation	Improved cardiac structure and function [109]
Fifteen minutes before ligation 0.15 mg/kg and 1.5 mg/kg body weight	LAD ligation	No changes in myocardial blood flow and infarct size [115]
Pre-treatment and post-treatment for 13 weeks 17 mg/kg body weight/day	LAD ligation	No changes in cardiac function and infarct size [116]

## Data Availability

Not applicable.
